# TGF-β Signaling Is Often Attenuated during Hepatotumorigenesis, but Is Retained for the Malignancy of Hepatocellular Carcinoma Cells

**DOI:** 10.1371/journal.pone.0063436

**Published:** 2013-05-21

**Authors:** Xiaoxin Mu, Shu Lin, Junhua Yang, Chen Chen, Yun Chen, Maryanne C. Herzig, Kenneth Washburn, Glenn A. Halff, Christi A. Walter, Beicheng Sun, Lu-Zhe Sun

**Affiliations:** 1 Liver Transplantation Center, The First Affiliated Hospital of Nanjing Medical University, Nanjing, China; 2 Department of Cellular and Structural Biology, University of Texas Health Science Center, San Antonio, Texas, United States of America; 3 Transplant Center, University of Texas Health Science Center, San Antonio, Texas, United States of America; 4 Cancer Therapy and Cancer Center, University of Texas Health Science Center, San Antonio, Texas, United States of America; 5 South Texas Veteran’s Health Care System, Audie Murphy Hospital, San Antonio, Texas, United States of America; Indiana University School of Medicine, United States of America

## Abstract

The role of transforming growth factor-beta (TGF-β) signaling in hepatocarcinogenesis remains controversial. We aimed to reveal TGF-β signaling status in human and murine tissues of hepatocellular carcinoma (HCC) and the mechanisms that mediate TGF-β’s role in regulating HCC malignancy. Here, TGF-β pathway component expression and activation in human and murine HCC tissues were measured with quantitative RT-PCR and Western blotting assays. The role of TGF-β receptor and Smad signaling in the growth and survival of several HCC cell lines was determined with several in vitro and in vivo approaches. We found that TGF-β receptor II (TβRII) expression was downregulated in two different HCC patient cohorts. Consistently, Smad3 phosphorylation was also downregulated in HCC tissues in comparison to that in adjacent normal tissues. Interestingly, many HCC cell lines were sensitive to TGF-β and growth-inhibited by exogenous TGF-β. However, stable knockdown of TβRII inhibited cell growth on plastic and in soft agar, and induced apoptosis resulting in suppressed subcutaneous tumor growth and metastatic potential in vivo. Furthermore, knockdown of Smad4 also led to a significant inhibition of growth on plastic and in soft agar with concomitant increase of apoptosis, PTEN expression, and reduced nuclear accumulation of linker region-phosphorylated Smad3. Taken together, TGF-β signaling pathway plays a dichotomous role in hepatocellular carcinogenesis. It appears to suppress HCC development, but is retained for HCC cell survival and malignancy. Furthermore, Smad4 can mediate both growth inhibitory activity induced by exogenous TGF-β and the survival activity induced by autocrine TGF-β revealing a delicate selection of the two opposing activities of TGF-β during HCC evolution.

## Introduction

Hepatocellular carcinoma (HCC), the major primary liver cancer, is the fifth most common cancer in men and women worldwide [1]. The mechanism underlying tumor initiation and progression of this disease is still not well understood, but partly due to deregulation of microenvironment homeostasis that involves the transforming growth factor β (TGF-β) signaling pathway [2], [3].

TGF-β isoforms are polypeptide cytokines. They are secreted in latent forms, which need to be activated to interact with cell surface receptors. Active homodimeric TGF-β isoforms initiate signaling by binding to the type I (RI) and type II (RII) TGF-β receptors, which contain an intracellular kinase domain. The activation of the RI kinase by ligand binding to the RII leads to the activation of Smad2 and Smad3 transcription factors via phosphorylation at their C-termini. The interaction between TGF-β and its receptors has also been shown to activate Smad-independent signaling pathways including PI3K/AKT and MAP kinase pathways. Because of this diverse array of signaling pathways activated by TGF-β, the role of TGF-β signaling in regulating cellular functions is often complex and context-dependent. TGF-β signaling through Smads is a well-known tumor suppressive pathway as it inhibits cellular proliferation by stimulating the expression of cyclin-dependent kinase inhibitors, p15 and p21, and induces apoptosis via various mechanisms [4]. On the other hand, TGF-β signaling has also been shown to drive tumor progression. This has been attributed to the activation of the Smad-independent pathways by TGF-β signaling in some cases [5].

In the normal liver, TGF-β is produced by nonparenchymal cells including sinusoidal endothelial cells, Kupffer cells, and lipocytes, and acts as a paracrine suppressor on the proliferation of normal hepatocytes[6]–[8]. However, TGF-β expression is often upregulated in transformed hepatocytes. In fact, it has been reported that plasma TGF-β was increased in HCC patients, especially during angiogenesis of HCC, and could be regarded as a marker for HCC progression [9], [10]. Most hepatocarcinoma cells are able to synthesize and secrete TGF-β continually by themselves. Interestingly, some reports showed low frequency of mutation of TGF-β receptor II (TβRII) and other TGF-β pathway genes in HCC, which are often found to be mutationally inactivated in other gastrointestinal cancers [11]–[15]. Thus, while TGF-β signaling is tumor-suppressive in various tissues, HCC cells often retain sensitivity to TGF-β and possess a functional autocrine TGF-β loop. However, the role of this autocrine TGF-β loop has not been well defined. A recent study showed that deletion of *Tgfbr2* in the setting of p53 loss reduced the formation of liver tumors, suggesting that TGF-β signaling was playing a promoting role in HCC induced by the loss of p53 [16]. However, knockout of TGF-β signaling components in other mouse tissues have in general promoted oncogene-induced tumor progression [2].

Because of the controversy surrounding the role of TGF-β signaling in hepatocytes and HCC cells, we have carried out comprehensive analyses of TGF-β pathway component expression and activation in human and murine HCC tissues and human HCC cell lines. The results shown below indicate a dichotomous role of TGF-β/Smad pathway during hepatocarcinogenesis. While the attenuation of TGF-β receptor signaling through Smad appears needed for the development of HCC, the attenuation appears limited and may even be reversed during the tumor progression for the survival of HCC cells. Our study further demonstrates that while HCC cells are growth-inhibited by exogenous TGF-β, they require autocrine TGF-β signaling for survival and malignancy, both of which are dependent on Smad4. As such, our study suggests a delicate balance of the two opposing activities of TGF-β during HCC evolution.

## Materials and Methods

### Human and Mouse Tissue Samples

Human HCC and corresponding adjacent tissues were obtained from patients undergoing surgical resection or liver transplantation at the Organ Transplant Center of the University of Texas Health Science Center at San Antonio and at the First Affiliated Hospital of Nanjing Medical University (Table S1). All the patients gave written informed consent and the study was also approved by the the Institutional Review Boards at the University of Texas Health Science Center at San Antonio and the First Affiliated Hospital of Nanjing Medical University. Mouse normal liver, adjacent to HCC, and HCC tissues were collected from C3HeB/FeJ mice, which spontaneously develop HCC as described previously [17]. All animal experiments were conducted following appropriate guidelines. They were approved by the Institutional Animal Care and Use Committee and monitored by the Department of Laboratory Animal Resources at the University of Texas Health Science Center at San Antonio.

### RNA Extraction, RT-PCR and Quantitative Real-time PCR

Total RNA was isolated from human tissues or HCC cell lines using Tri Reagent (Sigma-Aldrich, MO, USA) according to the manufacturer’s instructions. The extracted RNA was dissolved in DEPC-treated ddH_2_O and subjected to DNAse I treatment (Fisher Scientific, IL, USA) to remove genomic DNA contamination. DNAse I-treated total RNA (2 µg) was reverse-transcribed into cDNA using ABI high-capacity cDNA Reverse Transcription Kit. Quantitative real-time PCR was performed using Power SYBR Green PCR Mix (ABI) in Applied Biosystems. All primers used in this study (Table S2) were designed by Primer Premier 5.0 and synthesized by Integrated DNA Technologies (Coralville, IA).

### Chemical

Human recombinant TGF-β1 was dissolved in an aqueous solvent containing 4 mM HCl and 1 mg/ml bovine serum albumin (BSA). The TGF-β receptor I kinase inhibitor (RI-KI), also known as HTS466284 [18], was synthesized by the Chemical Synthesis Core of Vanderbilt University. The PI3K inhibitor, 2-(4-morpholinyl)-8-phenyl-4H-1-benzopyran-4-one, also known as LY294002, was purchased from Calbiochem (Billerica, MA).

### Protein Isolation and Western Blot Analysis

Proteins were collected from human tissue using T-PER Tissue Protein Extraction Reagent (Thermo Scientific, IL, USA) according to the manufacture’s protocol and whole cell lysates were prepared as described previously [19]. Whole cell extracts from mouse tissue were prepared as described previously [17]. Primary antibodies used were specific to p-Smad2 (pS465/467), p-Smad3 (pS423/425), T-Smad2, p-AKT, T-AKT, PTEN (Cell Signaling), p-Smad3L (p-s213) (Abcam), MSH2 (Oncogene), T-Smad3 (Zymed), p15, Smad4, TGF-βRI/II (Santa Cruz), GAPDH (Calbiochem, Billerica, MA).

### Cell Culture

Human HCC cell lines SNU398 (CRL-2233), SNU423 (CRL-223), HepG2 (HB-8065) and Sk-Hep-1 (HTB-52) were purchased from the American Type Culture collection (Manassas, VA). Huh7 cell line was kindly provided by Dr. Robert Lanford (Texas Biomedical Research Institute). All liver cancer cell lines were maintained in RPMI-1640 medium supplemented with 10% heat-inactivated fetal bovine serum, 1mM sodium pyruvate, 2.5 mg/ml glucose, and 0.5% penicillin/streptomycin. Cells were maintained in a humidified incubator at 37°C and 5% CO_2_.

### Luciferase Reporter Assay

Cells were plated at 1.5×10^5^ cell per well of a 12-well plate in triplicate 24 hours before transfection. pSBE4-Luc with repeated Smad binding elements and β-galactosidase expression plasmids were transiently co-transduced into cells by using LT-1 (Mirus Bio, Madison, WI) [20]. After 3 hours, the transfected cells were treated with/without 2 ng/ml TGF-β1 and/or 100 mM RI-KI. After additional 24 hours of incubation, cells were harvested and lysed as described previously [21]. Luciferase activity was assayed and normalized to β-galactosidase activity.

### MTT Assay

To determine the growth of HCC cell lines, cells were seeded in 96-well plates at 2,000 cells/well in the presence or absence of different concentrations of TGF-β1. 50 ul 3-(4,5-Dimethylthiazol-2-yl)-2,5-diphenyltetrazolium bromide (MTT) (2 mg/ml in sterile PBS ) was added into each well at indicated time point and cells were incubated at 37°C for 2 hours assays. 100 ul DMSO was added into each well after the medium was removed, and the plate was gently shaken on a shaker for 10 minutes. The absorbance was measured at 595 nm with a Microplate Reader (Bio Tek Instrument, Winooski, VT).

### Elisa Assay for TGF-β

Cells were plated at the same number in 24 well plates and fresh basic medium was changed when cells were exponentially growing at 70–80% confluence. After 24-hour incubation, the supernatants were collected, and cells were counted under microscope. The reading was normalized to cell number. This assay was performed by using the Duoset ELISA Development kit from R&D Systems (Minneapolis, MN) according to the manufacture’s protocol.

### Transfection and Determination of Knockdown of TGFBRII and Smad4

The TβRII shRNA, Smad4 shRNA and control shRNA in lentiviral vector pLK0.1-puro (Sigma, St Louis, MO, USA) were provided by Dr. John A. Copland. The sequence of shRNA is: TGFBRII: 5′-CCG GCC TGA CTT GTT GCT AGT CAT ACT CGA GTA TGA CTA GCA ACA AGT CAG GTT TTT G-3′ as described previously [22]; Smad4∶5′-CCG GCG AGT TGT ATC ACC TGG AAT TCT CGA GAA TTC CAG GTG ATA CAA CTC GTT TTT G-3′. The virus containing TβRII shRNA, Smad4 shRNA or control shRNA was produced by transfecting HEK 293FT packaging cells with shRNA expression plasmids in Lipofectamine 2000 according to the manufacture’s protocol. The virus was used to infect SNU423, Sk-Hep-1 and Huh7 cells with 8 µg/ml polybrene. Stable positive cells were selected in complete medium with 2 µg/ml (SNU423 and Sk-Hep-1) or 1 µg/ml (Huh7) puromycin. The control and TβRII knockdown SNU423 and Sk-Hep-1 cells were transduced with pLV411GeffLuc-flag-IRES-hrGFP (Luc-GFP) (a generous gift from Dr. Brian Rabinovich at MD Anderson Cancer Center) for stable expression of firefly luciferase (Luc) and the enhanced green fluorescent protein (GFP) for *in vivo* whole mouse imaging of metastasis. The knockdown of TβRII and Smad4 were confirmed with Western blotting and RT-PCR as described above.

### Soft and Hard Agarose Colony Formation Assay

Cells suspended in 0.5 ml of 0.4% or 1.2% low melting point agarose (Life Technologies, Carlsbad, CA, USA) with complete culture medium were gently seeded in 12-well plates coated with 0.8% agarose and 2 ng/ml TGF-β1 diluted in 250 µl medium was added on top when agarose was solidified as described previously [23]. Cell numbers were varied for different cell lines (SNU398, 4500 cells/well; SNU423, 6000 cells/well; HepG2, 4500 cells/well; Sk-Hep-1, 4500 cells/well; Huh7, 4500 cells/well). The plates were incubated at 37°C in tissue culture incubator for the indicated days. Colonies were stained with p-iodonitrotetrazolium violet (Sigma-Aldrich, St. Louis, MO) and counted by eye.

### Cell Death Detection ELISA

Cells were plated at 5×10^5^ cells/dish in 60 mm dishes and harvested after various treatments. Cell pellets were washed with cold 1× PBS twice, lysed with 40 µl Apoptosis Lysis Buffer and cell death was assayed using Cell Death Detection ELISA^PLUS^ (Roche Applied Science, Indianapolis, IN), which is based on the measurement of histone-complexed DNA fragments in the cytoplasm of apoptotic cells, according to the manufacture’s protocol.

### Annexin-V FITC Staining

Cells were plated at 5×10^5^ cells/dishes into 60 mm dishes. After reaching 70–80% confluence during exponential growth, cells were then starved for 48 hours when cells were exponentially growing at 70–80% confluence. Cells were harvested, washed with cold PBS and resuspended with binding buffer at a concentration of 2×10^6^ cell/ml. Cells were analyzed by using the Apo*Target*™ Annexin-V FITC Apoptosis kit (Invitrogen, Grand Island, NY) according to the manufacture’s protocol.

### Animal Experiments

Male athymic nude mice (Harlan Sprague Dawley, Inc., Indianapolis, IN), at 4–5 weeks of age, were used for *in vivo* animal experiments. The animals were maintained under specific pathogen-free conditions at the University of Texas Health Science Center, San Antonio, Texas. All animal protocols were approved and monitored by the Institutional Animal Care and Use Committee.

### *In vivo* Tumorigenicity and Metastasis Studies

To determine tumor growth *in vivo*, Sk-Hep-1/TβRIIshRNA/Luc-GFP and control cells were harvested from subconfluent exponentially growing cultures and inoculated subcutaneously in the rear back hindquarters of 4-week-old male nude mice. Cells was inoculated injected on both sides of each mouse at 3×10^6^ cells per 0.1 ml sterile PBS. Growth of xenografts was determined by measuring the volume (*V*), which was calculated with *V* = (*L*×*W* ^2^)×0.5, where *L* is the length and *W* is the width of the xenograft measured with a caliper. Mice were put through bioluminescence imaging to identify metastasis every two weeks as described previously [24]. After the termination of experiment at about 8 weeks, tumors were resected from anesthetized mice. Tumors were flash-frozen in liquid nitrogen for RNA extraction.

To determine the metastatic potential of the cells, a tail vein injection assay was performed. Sk-Hep-1/TβRII shRNA/Luc-GFP and control cells were harvested as above and resuspended at 0.5×10^6^ cells/0.1 ml sterile PBS. Cells were inoculated into mice by intravenous injection through tail vein. Bioluminenscence imaging was performed to monitor metastasis burden.

### Statistical Analysis

Differences between the means of control and experimental groups were analyzed by two-tailed Student’s t-tests. One-way ANOVA was used for the comparison among more than two groups. All statistical calculations were performed using the GraphPad Prism 5.0 software. P<0.05 was considered as significant.

## Results

### TGFβ Signaling is Down-regulated in Human and Mouse HCCs

**Figure 1:**
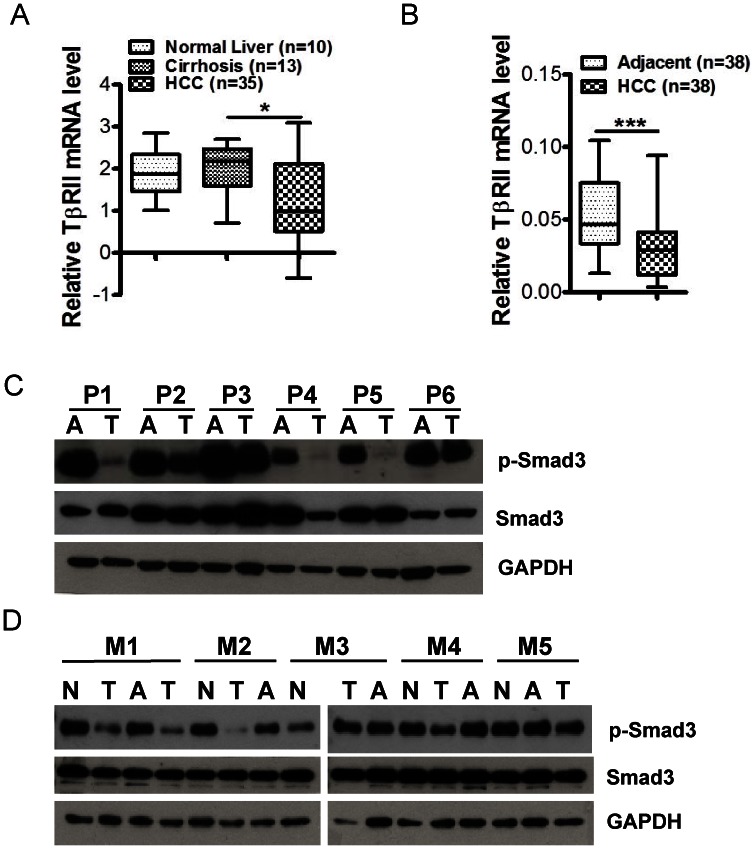
Down-regulation of TGFβ signaling pathway in human and mouse HCCs. (A) Significant decrease of TβRII expression in HCC compared with cirrhosis was seen in the report from Oncomine™. Shown are box-whisker plots of relative TβRII mRNA levels in human tissue. *, p<0.05 with one-way ANOVA. (B) Quantitative real-time PCR displayed significant reduction on TβRII expression in human HCC compared with precancerous tissue. Shown are box-whisker plots of the value of relative TβRII mRNA expression compared with GAPDH. ***, p<0.001. (C) Immunoblotting analysis of p-Smad3 and total Smad3 expression in paired human HCC (T) and adjacent tissues (A). (D) Immunoblotting analysis for p-Smad3 and total Smad3 expression in paired mouse HCC (T), Normal liver (N), and normal tissue adjacent to HCC (A). Mouse 1 had two live tumors.

To investigate the status of TGF-β signaling pathway, we initially analyzed gene expression profile studies in Oncomine™ (Compendia Bioscience, Ann Arbor, MI) and found that in the report by Wurmbach and co-workers [25], TβRII, one of the TGF-β signaling pathway components that is frequently dysregulated in cancer [26], was significantly reduced in HCCs when compared to that in cirrhosis (Fig. 1A). To confirm this finding, we examined TβRII expression level by performing quantitative real-time RT-PCR analysis. Thirty-eight pairs of normal tissues adjacent to HCC and HCC tissues were obtained from HCC patients at the time of surgical resection. The characteristics of the HCCs and the patient information are summarized in Table S1. Similarly, TβRII transcript levels in tumor tissues were also found to be significantly decreased in comparison with those in the adjacent normal tissues (Fig. 1B). Thus, TGF-β signaling activity is likely downregulated during the development of HCC, suggesting a tumor suppressive role of TGF-β signaling pathway.

Next, we measured the levels of phosphorylated Smad3 (P-Smad3) at its C-terminus as an indicator of TGF-β signaling activity in human and mouse HCCs by immunoblotting analysis. Interestingly, all six HCC had a modest to striking reduction of p-Smad3 in comparison to their adjacent normal tissues (Fig. 1C). The reduction of P-Smad3 in Patient 4 appears to be due to reduced total Smad3 level. Among five sets of mouse HCC tissues from C3HeB/FeJ male mice, which develop spontaneous HCC at a high frequency [17], we also observed a modest to striking decrease of p-Smad3 in the majority of HCCs (except Mouse 3) in comparison to the normal liver tissue and liver tissue adjacent to HCC (Fig. 1D). Thus, TGF-β signaling activity through the Smad pathway in both human and mouse HCCs appears down-regulated.

### TGF-β Signaling and Function in Human HCC Cell Lines

**Figure 2:**
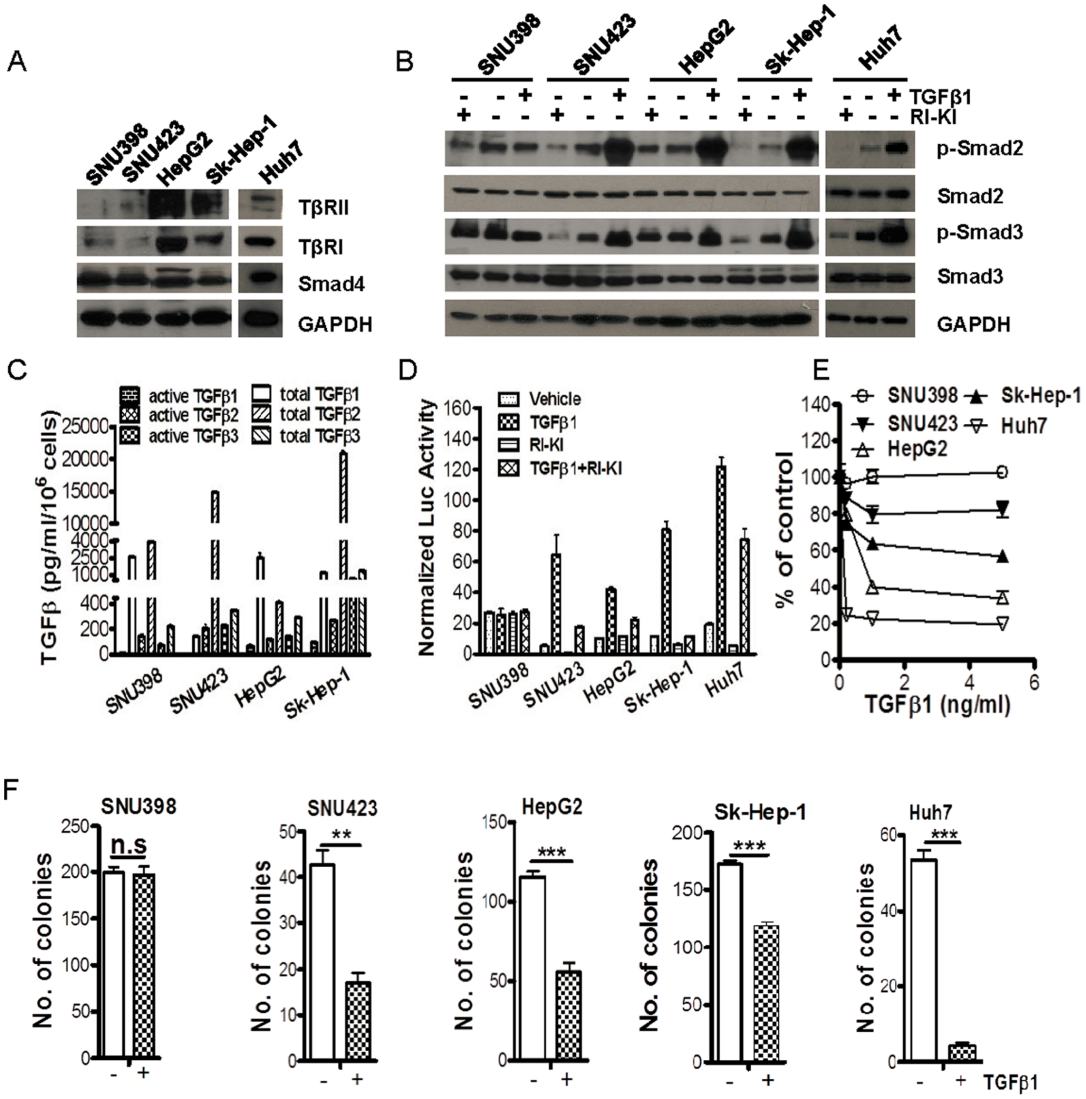
Status of TGF-β signaling pathway in five HCC cell lines. (A) Immunoblotting analysis for TβRII, TβRI and Smad4 in five HCC cell lines. (B) Immunoblotting analysis for Smad proteins in cell lysates of five HCC cell lines treated with or without 2 ng/ml TGF-β1 or 100 nM RI-KI for 1 hour. (C) Levels of the TGFβ isoforms in the media conditioned by four HCC cell lines were examined with the Duoset ELISA kit. Each data point represents mean ±SEM from three independent wells. (D) SBE-luciferase assay for TGF-β sensitivity in HCC cells. Cells were co-transfected with a TGF-β-responsive promoter-luciferase reporter (pSBE4-Luc) and a β-galactosidase (β-gal) expression plasmid and then treated with or without 2 ng/ml TGF-β1 or 100 nM RI-KI for 24 hours. Each data point represents mean±SEM from three independent transfections. Luciferase units in cell lysates were normalized to β-gal activity. (E) MTT assay for the effect of indicated doses of TGFβ1 on the proliferation of HCC cells after a 5-day treatment. Each data point represents mean±SEM from 4 independent wells. (F) soft-agar colony formation ability was assessed in HCC cells with or without 2 ng/ml TGFβ1 treatment for 14 days. Colonies in each culture well were counted after staining. Each data point represents mean±SEM from three independent wells. **, p<0.01; ***, p<0.001; “ns” means “no significance”.

To further investigate the role of TGF-β signaling pathway in human HCCs, we evaluated expression of several TGF-β signaling pathway components including TβRI, TβRII, and Smad4 in five HCC cell lines which have shown different TGF-β responsive characteristics [27]. Among these five cell lines, only SNU398 cell showed impaired TGF-β signaling pathway with little expression of TβRII when compared with other HCC cells (Fig. 2A). SNU423 cells also showed lower TβRI and TβRII expression whereas Sk-Hep-1, HepG2, and Huh7 cells showed higher expression (Fig. 2A). Furthermore, we determined the response of these five cell lines to TGF-β1 or RI-KI in regulating the phosphorylation of Smad2 and Smad3 by Western blotting analysis. All showed increased P-Smad2 and P-Smad3 in response to TGF-β1 except the SNU398 cell line. RI-KI treatment reduced basal P-Smad2 and P-Smad3 in SNU423, Sk-Hep-1 and Huh7 cells (Fig. 2B) suggesting that these cells possess autocrine TGF-β signaling activity. This notion is consistent with our findings that HCC cells produce detectable levels of all three TGF-β isoforms in the media conditioned by the cells (Fig. 2C). With a TGF-β responsive promoter-luciferase reporter assay, we observed that TGF-β1 stimulated luciferase activity in SNU423, HepG2, Sk-Hep-1 and Huh7 cells, whereas RI-KI significantly attenuated the activity in these cells (Fig. 2D). In contrast, there is no effect of TGF-β1 on luciferase activity in SNU398 cells. Similarly, as shown in Fig. 2E, TGF-β1 treatment induced various levels of growth inhibition in Huh7, HepG2, Sk-Hep-1, and SNU423 cells in a dose-dependent manner, but not in SNU398 cells. To evaluate the effect of TGF-β on *in vitro* tumorigenic ability of these HCC cell lines, we performed a soft-agar colony formation assay. Consistently, TGF-β1 attenuated colony formation ability of SNU423, HepG2, Sk-Hep-1 and Huh7 cells, but not SNU398 cell (Fig. 2F). Taken together, four of five HCC cell lines have an operational TGF-β/Smad signaling pathway and are growth inhibited by exogenous TGF-β1 to varying degrees in both two dimensional and three dimensional growth conditions.

### Abrogation of TGF-β Signaling Pathway Inhibits HCC Cell Growth and Promotes Apoptosis

**Figure 3:**
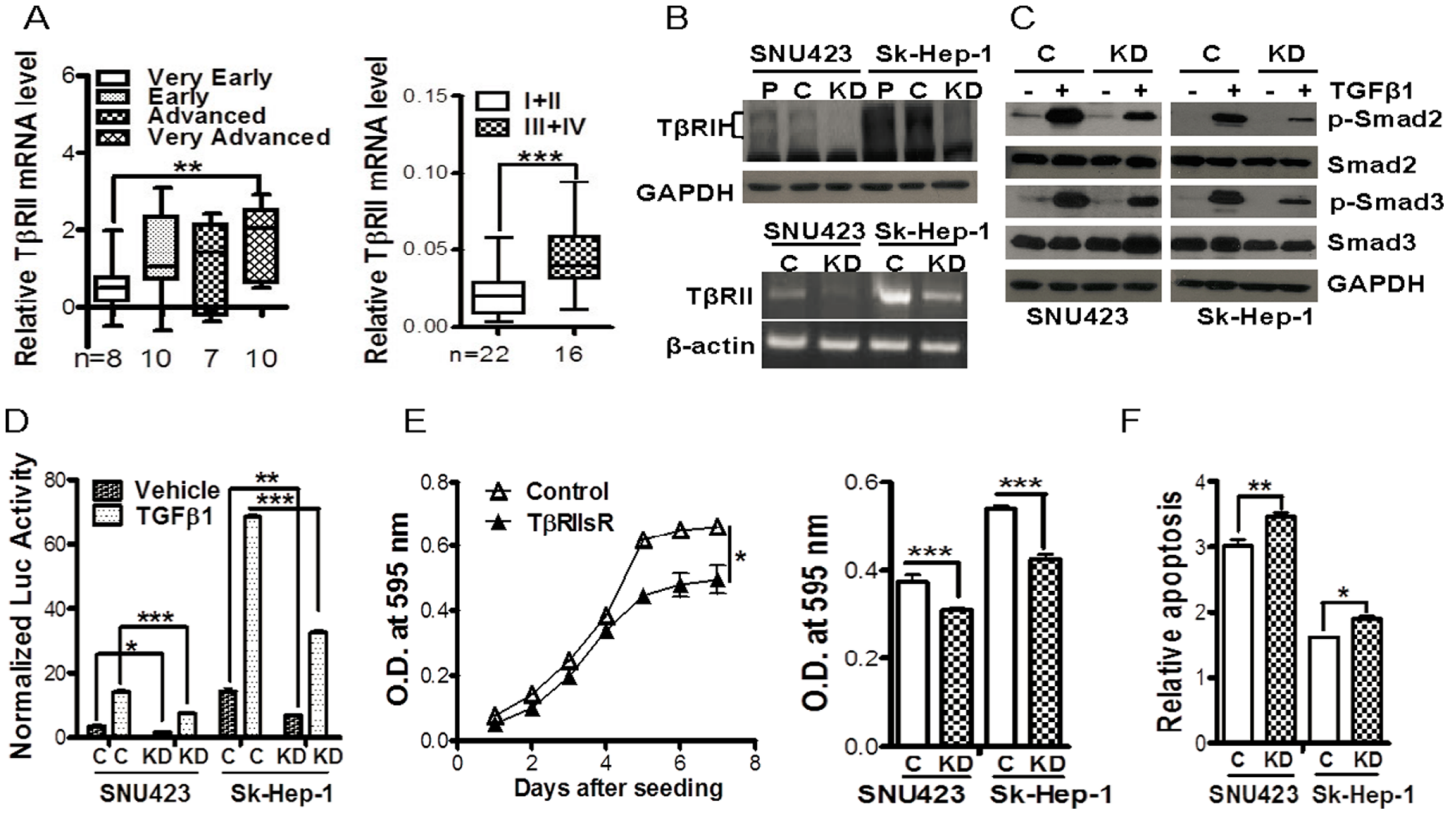
TβRII expression and its role in supporting HCC cell viability. (A) TβRII expression was compared in different stages of HCCs from ONCOMINE (left panel) and in 38 HCC tissue specimens by quantitative real-time RT-PCR (right panel). **, p<0.01; ***, p<0.001. (B) Western blotting analysis (upper panel) and RT-PCR (lower panel) showed decreased TβRII expression in TβRII knockdown HCC cells (KD) in comparison to the control vector-transfected cells (C) or parental cells (P). (C) SNU423 and Sk-Hep-1 cells with control and TβRII shRNA were treated with 2 ng/ml TGF-β1 for 1 hour. Immunoblotting analysis was performed for the levels of Smad proteins in cell lysates. (D) Control and TβRII shRNA cells were transfected with pSBE4-Luc and β-gal plasmids. Luciferase assay was done 24 hours after incubation with 2 ng/ml TGF-β1. β-gal-normalized luciferase units are presented as mean±SEM from three independent transfections. *, p<0.05; **, p<0.01; ***, p<0.001. (E) Cell proliferation was measured with MTT assay in control and TβRII-knockdown Sk-Hep-1 cells over a 7-day period (right panel) or on Day 5 after plating (left panel). Each data point represents mean ±SEM from four independent wells for Sk-Hep-1 and twelve independent wells for SNU423. A two-tailed paired T-test was performed to compare the two growth curves in the right panel. *, p<0.05; ***, p<0.001. (F) Effect of TβRII knockdown on apoptosis in SNU423 and Sk-Hep-1 cells. Cells were cultured in 60 mm dishes and harvested after 24-hour serum-deprivation at a density of 70–80% confluence. Each data point represents mean±SEM from three independent wells. *, p<0.05; **, p<0.01.

The above observations suggest that TβRII is a major target in the attenuation of TGF-β signaling activity during hepatocarcinogenesis and TGF-β treatment produced an apparent tumor suppressive activity in all HCC cell lines that are sensitive to TGF-β. Interestingly, by analyzing the reported gene profiling data by Wurmbach and co-workers, TβRII expression was found to be increased in very advanced HCCs when compared to very early HCCs (Fig. 3A) [25]. Similar phenomenon was also observed in our 38 HCC tissue specimens. TβRII expression in HCCs of Edmondson grade III/IV was significantly increased in comparison to that in HCCs of Edmondson grade I/II (Fig. 3A). To determine the role of TβRII and consequently that of the TGF-β signaling pathway in regulating the malignant phenotypes of HCC cells, we knocked down TβRII in SNU423 and Sk-Hep-1 cells with the stable expression of a TβRII shRNA as described previously [22]. Results indicated that knockdown of TβRII, confirmed by Western blotting analysis and RT-PCR (Fig. 3B), reduced both basal and TGF-β1-induced P-Smad2 and P-Smad3 (Fig. 3C), as well as Smad-responsive promoter activity as reported by luciferase activity (Fig. 3D), suggesting that autocrine TGF-β signaling is also abrogated by TβRII knockdown. More interestingly, the constitutive abrogation of autocrine TGF-β resulted in a significant inhibition of the growth of both cell lines as measured with MTT assays (Fig. 3E), as well as a significant stimulation of apoptosis as detected with an apoptosis ELISA (Fig. 3F). These results indicate that the autocrine TGF-β signaling does not inhibit the proliferation, instead is necessary for the viability of these HCC cells.

### Abrogation of TGF-β Signaling Attenuates the Anchorage-independent Colonogenic, Tumorigenic and Metastatic Potential of HCC Cells

**Figure 4:**
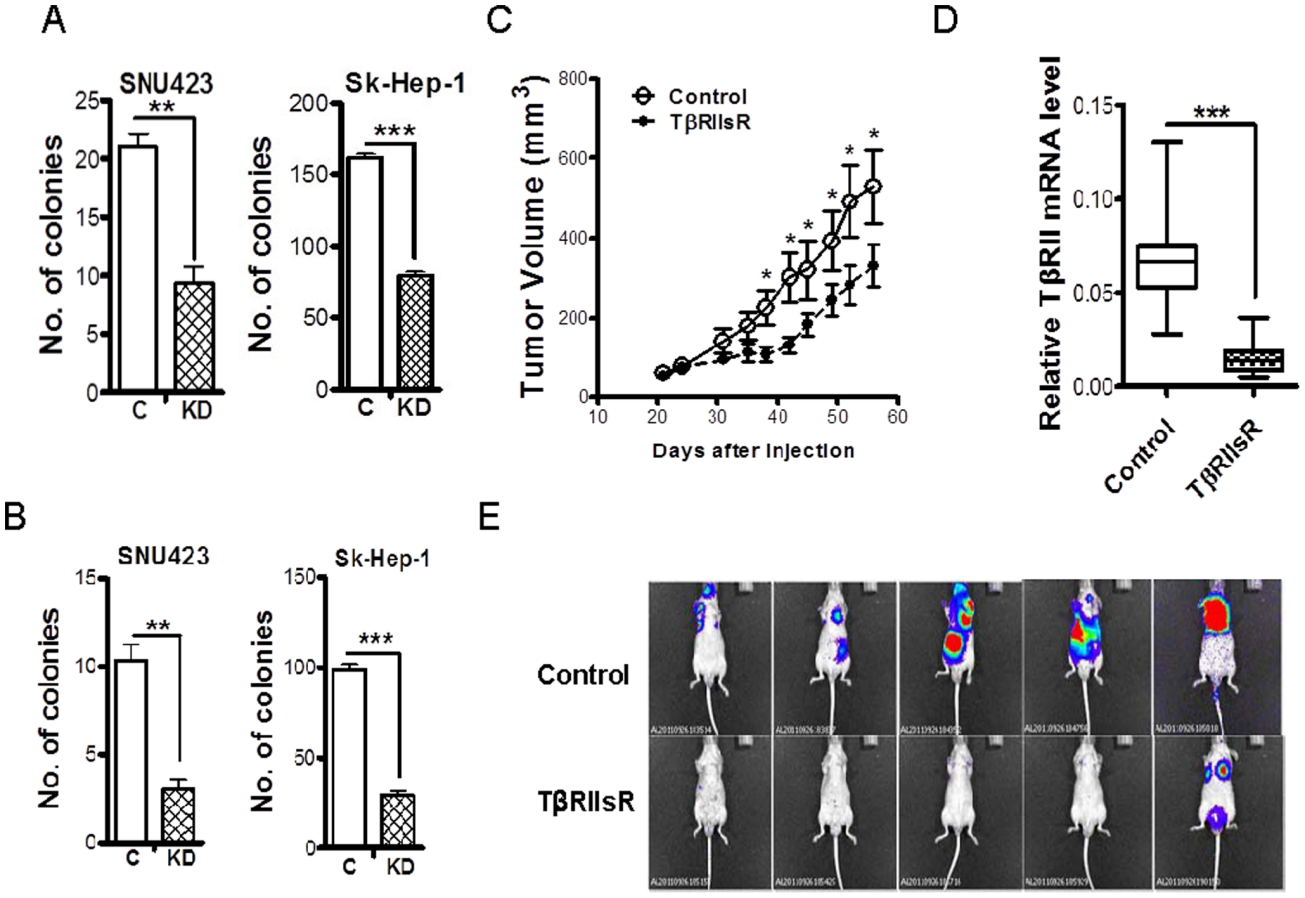
Reduced tumorigenic and metastatic ability in vitro and in vivo of SNU423 and Sk-Hep-1 cells by the knockdown of TβRII. **Soft-agar (A) or hard-agar (B) colony formation ability was assessed for SNU423 and Sk-Hep-1 cells with control or TβRII shRNA for 14 days. Colonies were counted after staining.** Each data point represents mean±SEM from three independent wells. **, p<0.01; ***, p<0.001. (C) Subcutaneous tumors were generated by Sk-Hep-1/control and Sk-Hep-1/TβRII shRNA cells. Each data point represents mean ±SEM from ten single tumors. *, p<0.05. (D) Reduced expression of TβRII mRNA in subcutaneous tumors formed by the Sk-Hep-1/TβRII shRNA cells was confirmed with quantitative real-time RT-PCR in comparison with that of Sk-Hep-1/control cell formed tumors. Total RNA was extracted from four tumors. ***, p<0.001. (E) Bioluminescence imaging showed reduced metastases incidence in the Sk-Hep-1/TβRII shRNA group in comparison with the control group.

We first determined the effect of TβRII knockdown on their in vitro tumorigenic potential with the anchorage-independent soft agar growth assay. We found that knockdown of TβRII significantly repressed the anchorage-independent growth ability of both SNU423 and Sk-Hep-1 cells when compared with their respective control cells (Fig. 4A). We also performed hard-agar colony formation assay, as it has been shown to predict the metastatic potential of tumor cells [23]. The results demonstrated that TβRII knockdown also reduced the colonogenic ability of both SNU423 and Sk-Hep-1 cells (Fig. 4B). These results suggest that TGF-β signaling is required for the maintenance of the malignancy of SNU423 and Sk-Hep-1 cells.

To confirm our in vitro findings, we next compared the growth of SNU423 and Sk-Hep-1 cells with or without TβRII knockdown in male athymic nude mice during a period of fifty-six days after inoculation of 3×10^6^ cells subcutaneously into rear hindquarters. Unfortunately, SNU423 cell did not form any tumors even after we inoculated a larger number of cells (5×10^6^ cells) mixed with matrigel, which has been shown to enhance the tumorigenicity of various transformed cells in nude mice [28], [29]. In contrast, Sk-Hep-1 control and TβRII shRNA cells started to form noticeable tumors 24 days after inoculation (Fig. 4C). Interestingly, the mean tumor growth rate in the TβRII shRNA group was initially much slower than that in the control group. As a result, the mean tumor volume of the two groups became significantly different during the late part of the experiment even though the mean tumor growth rates of the two groups eventually became similar (Fig. 4C). At the termination of the experiment, we harvested the tumors formed by the two cells and performed real-time RT-PCR to detect TβRII mRNA. As expected, TβRII shRNA tumors maintained the low expression of TβRII when compared with Sk-Hep-1/control tumors suggesting that the delayed increase of growth rate by TβRII shRNA tumors was not due to loss of TβRII knockdown (Fig. 4D).

Because the cell lines used for the in vivo experiments were stably transfected with a luciferase and GFP expression plasmid, we performed whole mouse bioluminescence imaging and also looked for GFP-expressing tumor cells in various visceral organs after they were excised from mice at the termination of the above experiment. No metastasis was observed with either imaging approach. Thus, to investigate how abrogation of TGF-β signaling may affect the *in vivo* metastatic potential of Sk-Hep-1 cells, we used an experimental metastasis model by inoculating the control and TβRII knockdown cells through tail vein. Metastasis induced by tumor cells was monitored by bioluminescence imaging every two weeks after inoculation. Consistent with the result from the hard-agar colony formation assay, the bioluminescence imaging taken four weeks after inoculation revealed that the knockdown of TβRII reduced the widespread dissemination of Sk-Hep-1 cell in nude mice (Fig. 4E). The incidence of significant metastasis in the Sk-Hep-1/control cell-inoculated mice was 100%, whereas in the Sk-Hep-1/TβRII shRNA cell-inoculated mice, it was only 20%.

### Smad Pathway Mediates Growth Inhibition by Exogenous TGF-β

**Figure 5:**
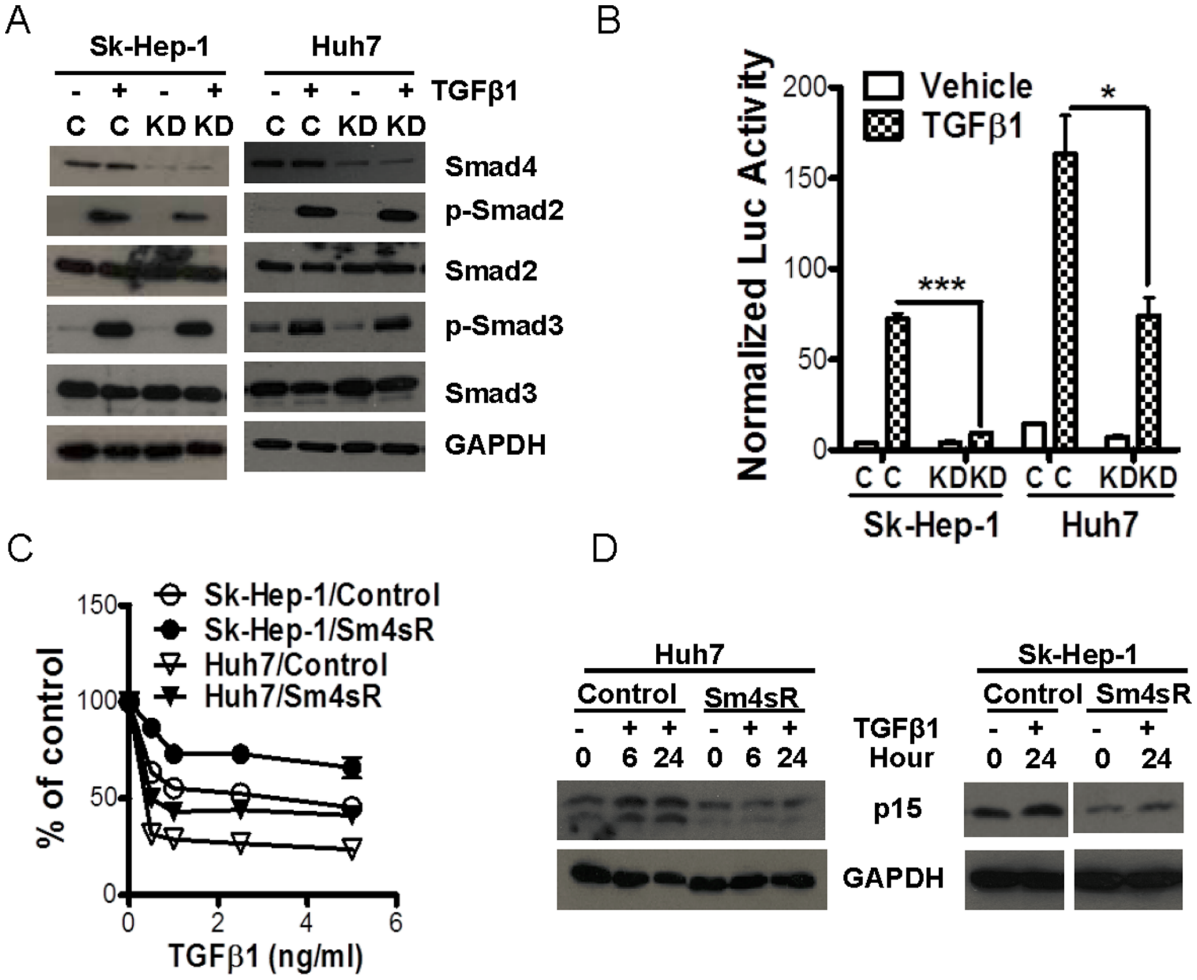
Smad-dependent inhibition of cell proliferation by TGF-β. (A) Immunoblotting analysis for Smad proteins and phosphorylated Smad2 and Smad3 was conducted in the cell lysates of control and Smad4-knockdown Sk-Hep-1 and Huh7 cells after treating the cells with TGF-β1 at 2 ng/ml for 1 hour. (B) SBE-luciferase assay was performed in Sk-Hep-1 and Huh7 cells transfected with or without Smad4 shRNA after TGFβ1 treatment as described in Fig. 2D. Each data point represents mean±SEM from three independent transfections. *, p<0.05; ***, p<0.001. (C) MTT assay was performed in Sk-Hep-1 and Huh7 cells with or without Smad4 knockdown after treatment with various concentrations of TGFβ1 for 5 days. (D) Immunoblotting analysis of p15 expression was carried with lysates from Sk-Hep-1 and Huh7 cells with or with Smad4 knockdown after treatment with TGFβ1 at 5 ng/ml for indicated time periods when the culture was 70–80% confluent.

TGF-β-induced growth inhibition is known to be mediated by the Smad pathway. On the other hand, it has also been shown to stimulate carcinoma cell survival by signaling through Smad-independent pathways [29]–[31]. As such, we hypothesized that abrogation of Smad pathway by knocking down Smad4 should attenuate TGF-β’s growth inhibitory activity while preserving the Smad-independent survival signaling of TGF-β, thus generating a different phenotype from that of the TβRII knockdown cells. As shown in Fig. 5A, expression of a Smad4 shRNA in Sk-Hep-1 and Huh7 cells reduced Smad4 protein levels in both cell lines. While the knockdown did not affect TGF-β-induced phosphorylation of Smad2 and Smad3, it led to a significant attenuation of TGF-β-induced Smad-responsive promoter activity (Fig. 5B) suggesting that Smad4 knockdown significantly attenuated Smad2/3/4 activity. Consistently, both Sk-Hep-1 and Huh7 cells were less sensitive to exogenous TGF-β-induced growth inhibition when their Smad4 was knocked down (Fig. 5C). Because the cyclin-dependent kinase inhibitors, p15 and p21, are major effectors of TGF-β-induced cell cycle arrest [32]; [33], we compared their expression in the control and Smad4 knockdown cells. While p21 expression was not detected in the two HCC cell lines, immunoblotting analysis showed that TGF-β-induced p15 expression was noticeably attenuated in both Huh7 and Sk-Hep-1 cells with Smad4 knockdown (Fig. 5D). These data demonstrate Smad4 is necessary for the growth inhibitory function of TGF-β in the HCC cells.

### Smad4 Supports HCC Cell Survival and Inhibits PTEN Expression

**Figure 6:**
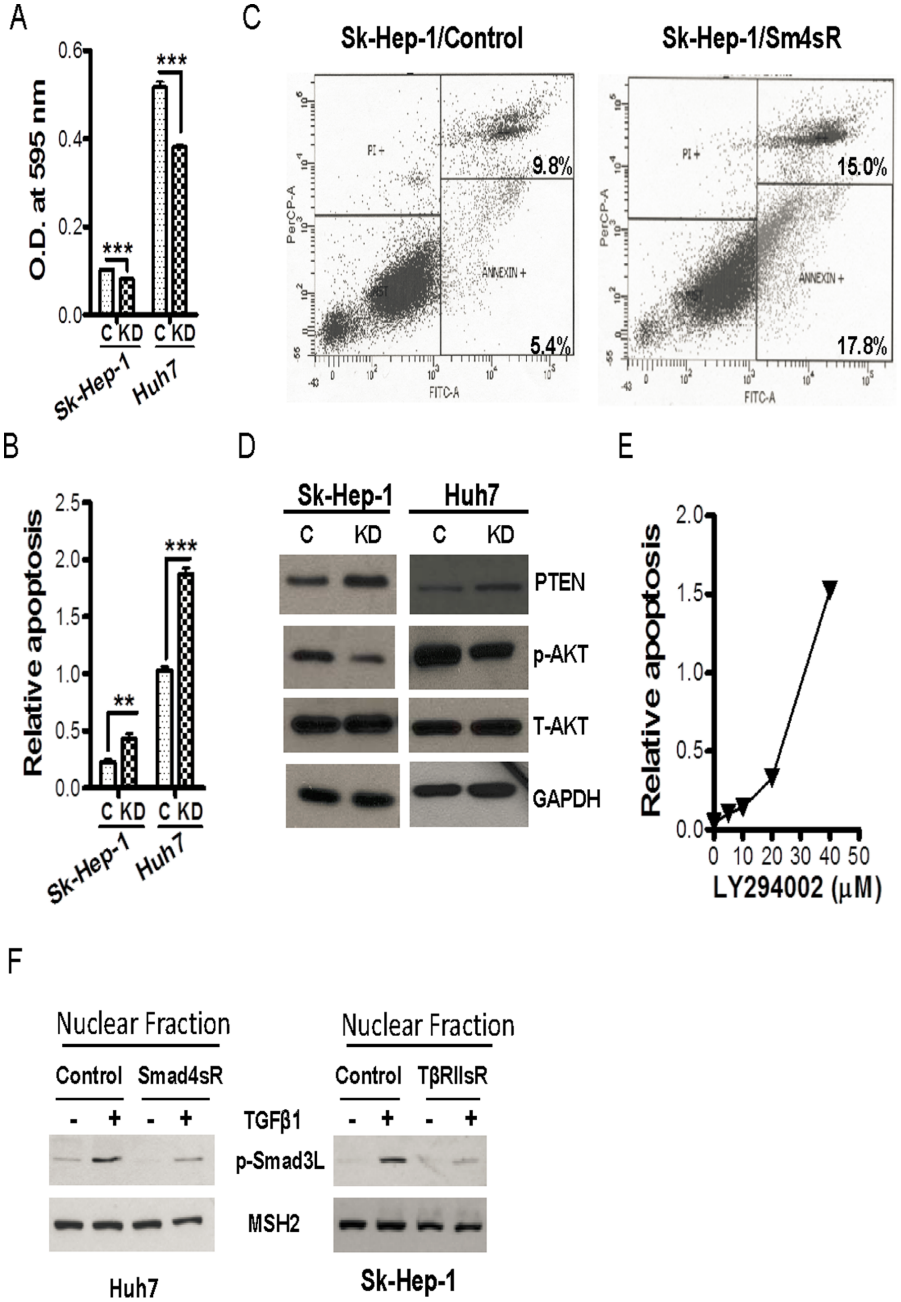
Reduced growth and increased apoptosis in HCC cells with Smad4 knockdown. (A) MTT assay for Sk-Hep-1 and Huh7 cells with control and Smad4 shRNA. **, p<0.01; ***, p<.001. (B) Apoptosis assay was performed in SK-Hep-1 and Huh7 cells with or without Smad4 shRNA using the Cell Death Detection ELISA. Each data point represents mean±SEM from three independent wells. **, p<0.01, ***, p<0.001. (C) Cell apoptosis was assessed in Sk-Hep-1 cell with or without Smad4 shRNA with Annexin-V FITC Apoptosis Kit. The cells in the lower right quarter are Annexin-V positive apoptotic cells as expressed with the percentage. (D) Immunoblotting analysis for indicated proteins in Sk-Hep-1 and Huh7 cells with or without Smad4 shRNA. (E) Cells were plated in a 6-well plate and treated with various concentrations of LY294002 for 24 hours when the culture was 70–80% confluence. Each data point represents mean ±SEM from three independent wells. (F) Immunoblotting analysis of P-Smad3L in the nuclei of Sk-Hep-1 and Huh7 with or without TβRII shRNA or Smad4 shRNA after treatment with TGF-β1 at 2 ng/ml for 1 hr.

**Figure 7:**
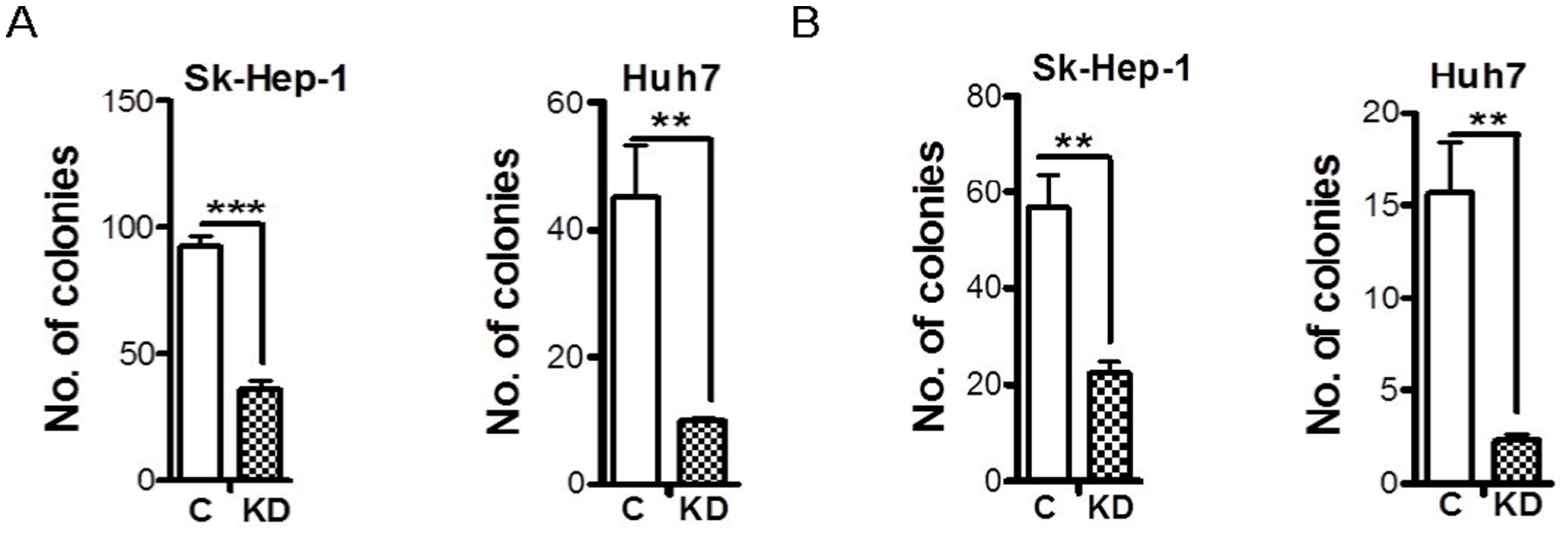
Attenuated potential of in vitro tumorigenic and metastatic potential of Sk-Hep-1 and Huh7 cells after Smad4 knockdown. Soft-agar (A) and hard-agar (B) colony formation ability was assessed in the control and Smad4 knockdown Sk-Hep-1 and Huh7 cells for 14 days. The colonies were stained and counted. Each data point represents mean±SEM from three independent wells. **, p<0.01; ***, p<0.001.

The confirmation of the growth inhibitory activity of the Smad pathway led us to expect that the Smad4 knockdown cells would grow faster and be more malignant than the control HCC cells. Thus, it was surprising to us that Smad4 knockdown in both Sk-Hep-1 and Huh7 cells led to significantly slower cell growth as detected with MTT assays (Fig. 6A) and significantly higher apoptosis as detected by both apoptosis ELISA (Fig. 6B) and Annexin-V staining assay (Fig. 6C). Thus, the knockdown of Smad4 produced identical phenotypes in the HCC cells as the knockdown of TβRII suggesting that the Smad pathway mediates both the growth inhibitory and cell survival activity of TGF-β signaling in the HCC cells. Because the tumor suppressor PTEN is downregulated in half of HCCs [34. and its expression is inhibited by both exogenous and autocrine TGF-β [19], [35], we next examined whether Smad signaling supports HCC cell survival by inhibiting PTEN expression. Indeed, the knockdown of Smad4 in both Sk-Hep-1 and Huh7 cells led to increased PTEN expression with a concomitant reduction of the active phospho-AKT (Fig. 6D). These results suggest that Smad pathway mediates TGF-β-induced suppression of PTEN. The increased PTEN expression and the decreased active AKT level in the Smad4 knockdown HCC cells likely contributed to the increased apoptosis as the treatment with an inhibitor of PI3K, the activator of AKT, also induced apoptosis in HCC cells (Fig. 6E). In addition, we also measured the levels of phosphorylated Smad3 at its link region (P-Smad3L) as a function of TGF-β signaling abrogation because nuclear P-Smad3L has been shown to have tumor-promoting activity [36]. We found that the levels of P-Smad3L in cytosol were hardly detectable in the HCC cell lines. In contrast, its level in the nucleus of the Smad4 knockdown Huh7 cells was decreased in comparison with that of the control cells in the absence or presence of TGF-β treatment (Fig. 6F). Similar phenomenon was also observed in TβRII knockdown Sk-Hep-1 cells (Fig. 6F). To determine the effect of silencing Smad4 on the tumorigenicity of Sk-Hep-1 and Huh7 cells, anchorage-independent colony formation assay was performed. As in the case of the abrogation of TGF-β signaling with the knockdown of TβRII, abrogation of Smad signaling in both Sk-Hep-1 and Huh7 cells also reduced their colony formation potential in both soft agar (Fig. 7A) and hard agar (Fig. 7B). Taken together, these results indicate that autocrine TGF-β/Smad signaling is indispensable for the survival and malignancy of HCC cells.

## Discussion

TGF-β signaling through its cell surface receptors and Smad proteins has been demonstrated as a tumor suppressive pathway in various types of cells, particularly in gastrointestinal malignancies. Among various receptors and Smad proteins that mediate TGF-β signaling, TβRII and Smad4 are most widely inactivated via gene mutation [14]. Although mutational inactivation of TGF-β/Smad pathway components is relatively rare in HCC [11]–[15], other mechanisms that abrogate the tumor suppressive function of TGF-β/Smad pathway have been reported. For example, TβRII expression was shown to be down regulated in HCC tissues in comparison with adjacent hepatic tissues [6], [37]. Both HBV and HCV associated HCC tissues were shown to have reduced level of phosphorylation of Smad3 at its C-terminus, which mediates its growth inhibitory activity [38], [39]. Consistent with these observations, our study also shows a significant downregulation of TβRII expression in HCC tissues and a widespread reduction of phospho-Smad3 at its C-terminus in both human and murine HCC tissues when compared to that in the adjacent hepatic tissues. Thus, our study further supports the tumor-suppressive concept of TGF-β pathway in hepatic tissue.

However, the controversy arises with respect to the role of TGF-β signaling pathway in transformed HCC cells. Owing to the relatively low inactivating mutations of the TGF-β receptor and Smad genes, many HCC cell lines retain an operational TGF-β signaling pathway, which was shown to mediate the growth inhibitory activity on plastic and in soft agar by exogenous TGF-β in our study. Others have shown that treatment with exogenous TGF-β induced cellular senescence and growth inhibition in various HCC cell lines in vitro and peritumoral injection of TGF-β inhibited the growth of tumors formed by Huh7 cells [40]. These observations appear to indicate that the tumor-suppressive activity of TGF-β is retained in various HCC cell lines. However, they do not address the role of autocrine TGF-β signaling in the control of malignant phenotypes of HCC cells. We sought to address this question by studying the effect of abrogation of autocrine TGF-β signaling via TβRII knockdown on the malignant properties of HCC cell lines. Our results indicate that the autocrine TGF-β signaling through its receptors is necessary for the survival and clonogenicity in suspension of both SNU423 and Sk-Hep-1 HCC cells that were used for TβRII knockdown experiments. The knockdown of TβRII also reduced the tumorigenic and metastatic properties of Sk-Hep-1 cells in vivo. Our observations are consistent with a recent report showing inhibition of hepatocarcinogenesis in hepatic specific knockout of p53 mice when *Tgfbr2* was deleted in hepatic tissue [16]. Others have shown that both exogenous and autocrine TGF-β signaling stimulated the proliferation of HCC-M and HCC-T cell lines [41], [42]. On the other hand, Senturk and co-workers reported that Hep3B-TR cells with deleted *TGFBR2* gene were much more tumorigenic than its parental Hep3B cells [40]. However, because Hep3B-TR cell line was established after long-term growth inhibitory selection of Hep3B cells with TGF-β treatment as a TGF-β resistant cell line [43], it is not known whether other genetic alterations and gene expression profile changes that are independent of TβRII loss as reported by Zimonjic and co-workers also contributed to its malignancy.

TGF-β signaling has been shown to be mediated by both Smad-dependent and Smad-independent pathways [2]. The latter includes MAPK and PI3K/AKT pathways, both of which have been shown to mediate mammary tumor cell survival and growth of TGF-β signaling [30], [31]. Because the tumor suppressive activity of TGF-β signaling is believed to be mediated by the Smad-dependent pathway and Smad4 plays a central role in TGF-β-induced Smad transcriptional activity, we knocked down Smad4 in HCC cells to elucidate the pathway(s) that mediates autocrine TGF-β/TβRII-induced cell survival. While knockdown of Smad4 did attenuate Smad transcriptional activity, and the potency of growth inhibition and p15 induction by exogenous TGF-β, it was surprising to find that Smad4 was also necessary for the survival of both Sk-Hep-1 and Huh-7 cells suggesting that the autocrine TGF-β-induced cell survival is at least in part mediated by the Smad pathway in these model systems. Autocrine TGF-β-induced growth inhibition of HCC-M and HCC-T cell lines was shown to be associated with its suppression of the promoter activity of p15 implicating the inhibition of p15 expression as a mechanism of growth promotion by TGF-β [41]. In contrast, we did not observe this phenomenon in Sk-Hep-1 and Huh-7 cells after knocking down Smad4. On the other hand, we observed an increase of PTEN expression and a decrease of phosphorylated/activated AKT in the Smad4 knockdown cells suggesting that the reduced AKT activity may contribute to the increased apoptosis and the reduced growth potential on plastic and in soft agar. Furthermore, as reviewed by Dr Matsuzaki, the activation of MAP kinases by growth factors including TGF-β can lead to phosphorylation of Smad2 and Smad3 at their linker region and p-Smad3L is involved in oncogenic signaling when translocated into the nucleus with Smad4 [36]. Interestingly, we found that knockdown of either TβRII or Smad4 attenuated TGF-β-induced nuclear accumulation of p-Smad3L suggesting that the tumor-promoting activity of autocrine TGF-β is likely mediated in part by its stimulation of linker region phosphorylation of Smad3. Further studies are needed to test these hypotheses.

In summary, our study together with others indicates that TGF-β signaling plays an important role in both suppression of HCC development and maintenance of malignant phenotypes of some HCCs. Its signaling strength appears to be finely tuned for its dichotomous actions during hepatocarcinogenesis. The Smad pathway appears to mediate the dual functions of TGF-β, likely in collaboration with different partners at different neoplastic stages. Further studies are needed to elucidate how Smad signaling network is altered during hepatocyte transformation to transduce a survival signal in HCC cells. Future studies will also determine whether HCC is uniquely suited for therapeutic intervention with novel TGF-β inhibitors since the Smad pathway in HCC cells is necessary for their survival rather than their growth inhibition.

## Supporting Information

Table S1 **Clinical Characteristics of the 38 HCC Patients.**
(DOC)

Table S2 **Primers used for quantitative real-time PCR and RT-PCR.**
(DOC)

## References

[1] Jemal A, Bray F (2011) Center MM, Ferlay J, Ward E, et al (2011) Global cancer statistics. CA Cancer J Clin 61: 69–90. 21296855 10.3322/caac.20107

[2] Massague J (2008) TGFbeta in Cancer. Cell 134: 215–30. 18662538 10.1016/j.cell.2008.07.001 PMC3512574

[3] Siegel PM, Massague J (2003) Cytostatic and apoptotic actions of TGF-beta in homeostasis and cancer. Nat Rev Cancer 3: 807–21. 14557817 10.1038/nrc1208

[4] Massague J (2004) G1 cell-cycle control and cancer. Nature 432: 298–306. 15549091 10.1038/nature03094

[5] Moustakas A, Heldin CH (2005) Non-Smad TGF-beta signals. J Cell Sci 118: 3573–84. 16105881 10.1242/jcs.02554

[6] Bedossa P, Peltier E, Terris B, Franco D, Poynard T (1995) Transforming growth factor-beta 1 (TGF-beta 1) and TGF-beta 1 receptors in normal, cirrhotic, and neoplastic human livers. Hepatology 21: 760–6. 7875675

[7] Bissell DM, Wang SS, Jarnagin WR, Roll FJ (1995) Cell-specific expression of transforming growth factor-beta in rat liver. Evidence for autocrine regulation of hepatocyte proliferation. J Clin Invest 96: 447–55. 7615817 10.1172/JCI118055 PMC185218

[8] Fausto N, Laird AD, Webber EM (1995) Liver regeneration. 2. Role of growth factors and cytokines in hepatic regeneration. FASEB J 9: 1527–36. 8529831 10.1096/fasebj.9.15.8529831

[9] Ito N, Kawata S, Tamura S, Shirai Y, Kiso S, et al (1995) Positive correlation of plasma transforming growth factor-beta 1 levels with tumor vascularity in hepatocellular carcinoma. Cancer Lett 89: 45–8. 7882301 10.1016/0304-3835(95)90156-6

[10] Shirai Y, Kawata S, Tamura S, Ito N, Tsushima H, et al (1994) Plasma transforming growth factor-beta 1 in patients with hepatocellular carcinoma. Comparison with chronic liver diseases. Cancer 73: 2275–9. 7513247 10.1002/1097-0142(19940501)73:9<2275::aid-cncr2820730907>3.0.co;2-t

[11] Kawate S, Ohwada S, Hamada K, Koyama T, Takenoshita S, et al (2001) Mutational analysis of the Smad6 and Smad7 genes in hepatocellular carcinoma. Int J Mol Med 8: 49–52. 11408948

[12] Kawate S, Takenoshita S, Ohwada S, Mogi A, Fukusato T, et al (1999) Mutation analysis of transforming growth factor beta type II receptor, Smad2, and Smad4 in hepatocellular carcinoma. Int J Oncol 1999 Jan 14: 127–31. 10.3892/ijo.14.1.127 9863018

[13] Yakicier MC, Irmak MB, Romano A, Kew M, Ozturk M (1999) Smad2 and Smad4 gene mutations in hepatocellular carcinoma. Oncogene 18: 4879–83. 10490821 10.1038/sj.onc.1202866

[14] Levy L, Hill CS (2006) Alterations in components of the TGF-beta superfamily signaling pathways in human cancer. Cytokine Growth Factor Rev 17: 41–58. 16310402 10.1016/j.cytogfr.2005.09.009

[15] Furuta K, Misao S, Takahashi K, Tagaya T, Fukuzawa Y, et al (1999) Gene mutation of transforming growth factor beta1 type II receptor in hepatocellular carcinoma. Int J Cancer 81: 851–3. 10362128 10.1002/(sici)1097-0215(19990611)81:6<851::aid-ijc2>3.0.co;2-d

[16] Morris SM, Baek JY, Koszarek A, Kanngurn S, Knoblaugh SE, et al (2012) Transforming growth factor-beta signaling promotes hepatocarcinogenesis induced by p53 loss. Hepatology 55: 121–31. 21898503 10.1002/hep.24653 PMC3237853

[17] Zhou ZQ, Manguino D, Kewitt K, Intano GW, Mcmahan CA, et al (2001) Spontaneous hepatocellular carcinoma is reduced in transgenic mice overexpressing human O6- methylguanine-DNA methyltransferase. Proc Natl Acad Sci U S A 98: 12566–71. 11606727 10.1073/pnas.221232998 PMC60094

[18] Singh J, Chuaqui CE, Boriack-Sjodin PA, Lee WC, Pontz T, et al (2003) Successful shape-based virtual screening: the discovery of a potent inhibitor of the type I TGFbeta receptor kinase (TbetaRI). Bioorg Med Chem Lett 13: 4355–9. 14643325 10.1016/j.bmcl.2003.09.028

[19] Lei X, Bandyopadhyay A, Le T, Sun L (2002) Autocrine TGFbeta supports growth and survival of human breast cancer MDA-MB-231 cells. Oncogene 21: 7514–23. 12386814 10.1038/sj.onc.1205966

[20] Zawel L, Dai JL, Buckhaults P, Zhou S, Kinzler KW, et al (1998) Human Smad3 and Smad4 are sequence-specific transcription activators. Mol Cell 1: 611–7. 9660945 10.1016/s1097-2765(00)80061-1

[21] Chen C, Wang XF, Sun L (1997) Expression of transforming growth factor beta (TGFbeta) type III receptor restores autocrine TGFbeta1 activity in human breast cancer MCF-7 cells. J Biol Chem 272: 12862–7. 9139748 10.1074/jbc.272.19.12862

[22] Lin S, Yu L, Yang J, Liu Z, Karia B, et al (2011) Mutant p53 disrupts role of ShcA protein in balancing Smad protein-dependent and -independent signaling activity of transforming growth factor-beta (TGF-beta). J Biol Chem 286: 44023–34. 22039050 10.1074/jbc.M111.265397 PMC3243540

[23] Li L, Price JE, Fan D, Zhang RD, Bucana CD, et al (1989) Correlation of growth capacity of human tumor cells in hard agarose with their in vivo proliferative capacity at specific metastatic sites. J Natl Cancer Inst 81: 1406–12. 2778827 10.1093/jnci/81.18.1406

[24] Mishra S, Tang Y, Wang L, deGraffenried L, Yeh IT, et al (2011) Blockade of transforming growth factor-beta (TGFbeta) signaling inhibits osteoblastic tumorigenesis by a novel human prostate cancer cell line. Prostate 71: 1441–54. 21321980 10.1002/pros.21361 PMC3108007

[25] Wurmbach E, Chen YB, Khitrov G, Zhang W, Roayaie S, et al (2007) Genome-wide molecular profiles of HCV-induced dysplasia and hepatocellular carcinoma. Hepatology 45: 938–47. 17393520 10.1002/hep.21622

[26] Bierie B, Moses HL (2006) TGF-beta and cancer. Cytokine Growth Factor Rev 17: 29–40. 16289860 10.1016/j.cytogfr.2005.09.006

[27] Coulouarn C, Factor VM, Thorgeirsson SS (2008) Transforming growth factor-beta gene expression signature in mouse hepatocytes predicts clinical outcome in human cancer. Hepatology 47: 2059–67. 18506891 10.1002/hep.22283 PMC2762280

[28] Gourley C, Paige AJ, Taylor KJ, Scott D, Francis NJ, et al (2005) WWOX mRNA expression profile in epithelial ovarian cancer supports the role of WWOX variant 1 as a tumour suppressor, although the role of variant 4 remains unclear. Int J Oncol 26: 1681–9. 15870886 10.3892/ijo.26.6.1681 PMC4166600

[29] Fridman R, Giaccone G, Kanemoto T, Martin GR, Gazdar AF, et al (1990) Reconstituted basement membrane (matrigel) and laminin can enhance the tumorigenicity and the drug resistance of small cell lung cancer cell lines. Proc Natl Acad Sci U S A 87: 6698–702. 2168554 10.1073/pnas.87.17.6698 PMC54604

[30] Muraoka-Cook RS, Shin I, Yi JY, Easterly E, Barcellos-Hoff MH, et al (2006) Activated type I TGFbeta receptor kinase enhances the survival of mammary epithelial cells and accelerates tumor progression. Oncogene 25: 3408–23. 16186809 10.1038/sj.onc.1208964

[31] Lei X, Yang J, Nichols RW, Sun LZ (2007) Abrogation of TGFbeta signaling induces apoptosis through the modulation of MAP kinase pathways in breast cancer cells. Exp Cell Res 313: 1687–95. 17382930 10.1016/j.yexcr.2007.02.016 PMC1905831

[32] Hannon GJ, Beach D (1994) p15INK4B is a potential effector of TGF-beta-induced cell cycle arrest. Nature 371: 257–61. 8078588 10.1038/371257a0

[33] Datto MB, Li Y, Panus JF, Howe DJ, Xiong Y, et al (1995) Transforming growth factor beta induces the cyclin-dependent kinase inhibitor p21 through a p53-independent mechanism. Proc Natl Acad Sci U S A 92: 5545–9. 7777546 10.1073/pnas.92.12.5545 PMC41732

[34] Fabregat I, Roncero C, Fernandez M (2007) Survival and apoptosis: a dysregulated balance in liver cancer. Liver Int 27: 155–62. 17311609 10.1111/j.1478-3231.2006.01409.x

[35] Li DM, Sun H (1997) TEP1, encoded by a candidate tumor suppressor locus, is a novel protein tyrosine phosphatase regulated by transforming growth factor beta. Cancer Res 57: 2124–9. 9187108

[36] Matsuzaki K (2011) Smad phosphoisoform signaling specificity: the right place at the right time. Carcinogenesis 32: 1578–88. 21798854 10.1093/carcin/bgr172 PMC3204345

[37] Kiss A, Wang NJ, Xie JP, Thorgeirsson SS (1997) Analysis of transforming growth factor (TGF)-alpha/epidermal growth factor receptor, hepatocyte growth Factor/c-met,TGF-beta receptor type II, and p53 expression in human hepatocellular carcinomas. Clin Cancer Res 3: 1059–66. 9815784

[38] Murata M, Matsuzaki K, Yoshida K, Sekimoto G, Tahashi Y, et al (2009) Hepatitis B virus X protein shifts human hepatic transforming growth factor (TGF)-beta signaling from tumor suppression to oncogenesis in early chronic hepatitis B. Hepatology. 49: 1203–17. 10.1002/hep.22765 19263472

[39] Matsuzaki K, Murata M, Yoshida K, Sekimoto G, Uemura Y, et al (2007) Chronic inflammation associated with hepatitis C virus infection perturbs hepatic transforming growth factor beta signaling, promoting cirrhosis and hepatocellular carcinoma. Hepatology 46: 48–57. 17596875 10.1002/hep.21672

[40] Senturk S, Mumcuoglu M, Gursoy-Yuzugullu O, Cingoz B, Akcali KC, et al (2010) Transforming growth factor-beta induces senescence in hepatocellular carcinoma cells and inhibits tumor growth. Hepatology 52: 966–74. 20583212 10.1002/hep.23769

[41] Matsuzaki K, Date M, Furukawa F, Tahashi Y, Matsushita M, et al (2000) Autocrine stimulatory mechanism by transforming growth factor beta in human hepatocellular carcinoma. Cancer Res 60: 1394–402. 10728705

[42] Matsuzaki K, Date M, Furukawa F, Tahashi Y, Matsushita M, et al (2000) Regulatory mechanisms for transforming growth factor beta as an autocrine inhibitor in human hepatocellular carcinoma: implications for roles of smads in its growth. Hepatology 32: 218–27. 10915727 10.1053/jhep.2000.9145

[43] Inagaki M, Moustakas A, Lin HY, Lodish HF, Carr BI (1993) Growth inhibition by transforming growth factor beta (TGF-beta) type I is restored in TGF-beta-resistant hepatoma cells after expression of TGF-beta receptor type II cDNA. Proc Natl Acad Sci U S A 90: 5359–63. 8389483 10.1073/pnas.90.11.5359 PMC46716

