# Supplementary material for: TGF-β Signaling Is Often Attenuated during Hepatotumorigenesis, but Is Retained for the Malignancy of Hepatocellular Carcinoma Cells
Source: PLoS One. 2013 May 21;8(5):e63436. doi: 10.1371/journal.pone.0063436 (PMC3660330; doi:10.1371/journal.pone.0063436)
Supplement: Table S1 — Clinical Characteristics of the 38 HCC Patients. (DOC) [file pone.0063436.s001.doc]

**Table S1.** Clinical Characteristics of the 38 HCC Patients

| **Patients Characteristics**  **Patients** |
| --- |
| No. of patients 38  Age, year (median, range) 52, (29-80)  Gender (male/female) 33/5  Hepatitis virus infection (negative/HBV/HCV/co-infection) 6/31/0/1  Cirrhosis (absent/present) 18/20  AFP, µg/L (≤13.6/>13.6) 14/24  Tumor size, cm (≤5/>5) 8/30  Tumor multiplicity (solitary/multiple) 15/23  Vascular invasion (absent/present) 34/4  Edmondson grade (I+II/III+IV) 22/16 |
