# Supplementary material for: TGF-β Signaling Is Often Attenuated during Hepatotumorigenesis, but Is Retained for the Malignancy of Hepatocellular Carcinoma Cells
Source: PLoS One. 2013 May 21;8(5):e63436. doi: 10.1371/journal.pone.0063436 (PMC3660330; doi:10.1371/journal.pone.0063436)
Supplement: Table S2 — Primers used for quantitative real-time PCR and RT-PCR. (DOC) [file pone.0063436.s002.doc]

**Table S2. Primers used for quantitative real-time PCR** and RT-PCR

| **Human Gene Sequence 5’-3’ Product size (bp)** |
| --- |
| **Quantitative real-time PCR** |
| TGFβRII Sense 5’-CCTGCGTCTGGACCCTACT-3’ 54  Anti-sense 5’-ACCTGCCCACTGTTAGCC-3’  GAPDH Sense 5’-GACCTGACCTGCCGTCTA-3’ 148  Anti-sense 5’-AGGAGTGGGTGTCGCTGT-3’ |
| **RT-PCR** |
| TGFβRII Sense 5’-AGCTGGGCAGCTCCCTCGCC-3’ 493  Anti-sense 5’-CCCACTGTTAGCCAGGTCATCCACA-3’  GAPDH Sense 5'-CCGCCAGCTCACCATGGATGAT-3' 159  Anti-sense 5'-TGACCCATGCCCACCATCAC-3' |
